# Intrinsic structural distortion and exchange interactions in SmFe_*x*_Cr_1−*x*_O_3_ compounds†

**DOI:** 10.1039/c7ra13615c

**Published:** 2018-02-27

**Authors:** Zhongcheng Xiang, Wenping Li, Yimin Cui

**Affiliations:** Key Laboratory of Micro-nano Measurement-Manipulation and Physics, Ministry of Education, Beihang University Beijing 100191 China cuiym@buaa.edu.cn

## Abstract

The effect of substituting different amounts of magnetic metal Fe on the magnetic properties of SmFe_*x*_Cr_1−*x*_O_3_ (0 < *x* < 0.5) is reported here in order to probe the relation between the structural distortion and magnetism in these materials. The structural properties of the samples were characterized using X-ray diffraction with Rietveld refinements, and Raman spectroscopy carried out at ambient temperature. Magnetization data reveals the Neel temperature (*T*_N_, where the Cr(Fe) ions order) increases with an increase in the average B-site ionic radius, and average Cr(Fe)–O–Cr(Fe) bond angle. By fitting the temperature dependence of the magnetic susceptibility to the Curie–Weiss law modified by the Dzyaloshinskii–Moriya (DM) interaction, the strengths of the symmetric and antisymmetric Cr(Fe)–Cr(Fe) exchange interactions (*J* and *D*) were determined. It was found that the strength of the symmetric interaction *J* (reflected in the changes in the Neel temperature) increases with the replacement of Cr^3+^ with Fe^3+^, which is ascribed to the changes in the average Cr(Fe)–O–Cr(Fe) bond angle and bond lengths. Meanwhile, the antisymmetric interaction *D* a slightly decreases, which may be ascribed to the displacement of oxygen ions (dO) away from their “original” middle point.

## Abstract 2

The relationship between intrinsic structural distortions and exchange interactions in SmFe_*x*_Cr_1−*x*_O_3_ compounds was studied.

## Graphical abstract

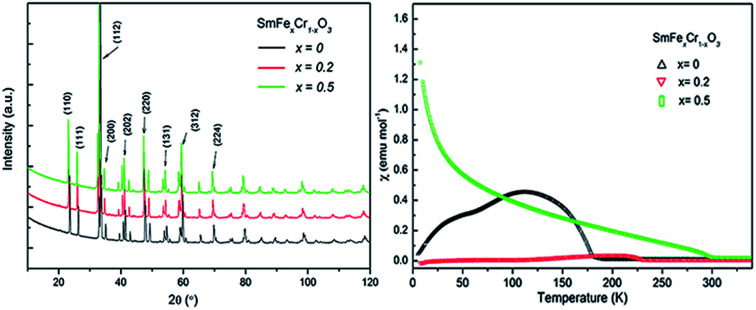

## Introduction

Rare-earth (R) transition metal (M) orthorhombic perovskite materials with ABO_3_-type perovskite structures, such as orthochromites RCrO_3_, and orthoferrites RFeO_3_, have received great interest, and possess more than one ferroic order, for example, ferroelectricity (FE), ferromagnetism (FM), and ferroelasticity.^1,2^ Perovskite structures can demonstrate a variety of functions because of the wide selection of elements they can incorporate, driving structural distortions that influence the magnetic and electronic properties, and magnetoelectric properties.^3^ The structure can also accommodate various types of disorder such as point substitutions or vacancies in some cases. Element substitution in the A-site and B-site of rare-earth orthorhombic perovskite materials leads to changes in the average B–O–B bond angle and bond lengths. Naturally, the perovskite materials will suffer from structural deformation or tilting. Such tilting controls both the magnetic superexchange, *J*, and the orbital overlap between B and O ions, which thus determines the magnetic ordering temperature and the conductivity.^4–6^ It has been widely accepted that the B–O–B bond angle (B = transition metal), which is reduced from 180° due to the cooperative octahedral-site rotations in the orthorhombic perovskite, is a major factor in determining the B–O–B superexchange interaction. The empirical relationship between the superexchange coupling *J* and the superexchange angle for the RFeO_3_ family has been reported from *J* ∼ cos*θ* (ref. 7) to *J* ∼ cos^2^*θ* (ref. 8) and finally to *J* ∼ cos^4^ ((π − θ)/2)/*d*^7^ (ref. 9), where *θ* and *d* denote the average B–O–B bond angle and the average B–O bond length, respectively.

In addition, RCrO_3_ and RFeO_3_ with ABO_3_-type perovskite structures exhibit antiferromagnetism with a weak ferromagnetic moment (WFM) due to the canted nature of the Cr^3+^ and Fe^3+^ moments below the Neel temperatures.^10,11^ In recent publications,^12,13^ the weak ferromagnetism of various antiferromagnetic compounds can mainly be explained by the Dzyaloshinskii–Moriya interaction, and the Hamiltonian can be expressed as 
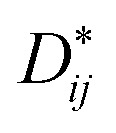
(*S*_*i*_ × *S*_*j*_). For a perovskite structure with bent B–O–B bonds, the vector *D*_*ij*_ must be perpendicular to the B–O–B plane and determined by the symmetry restrictions.^14,15^ In other words, substitution in the A-site and B-site and a size mismatch between the A and B ions usually make the oxygen octahedra tilt and rotate, resulting in a distortion. Therefore, each oxygen ion sandwiched between two neighboring B ions may move away from the middle point, giving a bent B–O–B bond and breaking the B–B axis rotation symmetry. This bent B–O–B bond will change the DM interaction as a relativistic correction to the superexchange between the magnetic B ions. Through the above analysis, the strengths of the symmetric and antisymmetric B–B exchange interactions (*J* and *D*) are changed by structural deformation and tilting, which is caused by element substitution in perovskite materials.

Interestingly, SmCrO_3_ is a representative rare-earth orthochromite that has been believed to possess canted G-type antiferromagnetism with a magnetic ordering temperature around 192 K.^16^ In recent years, it has been believed that a variety of interesting properties can be achieved by alloying different kinds of cation at the B-site of perovskite materials. According to the Goodenough–Kanamori theory, Fe^3+^ is the best choice for substituting Cr^3+^ in order to show superior magnetic properties, due to superexchange interactions.^17^ Studies on AB_*x*_B′_1−*x*_O_3_ perovskite compounds have shown that the structures and physical properties of these compounds strongly depend on the ionic size and charge differences of the B-site cations (B and B′).^18,19^

In this paper, polycrystalline samples SmFe_*x*_Cr_1−*x*_O_3_ (0 < *x* < 0.5) were compounded *via* a solid state reaction, and their structures were confirmed using XRD and Raman spectroscopy techniques, which reveal the evolution of the distorted perovskite structure with Fe substitution. The temperature dependent dc magnetic measurements reveal obvious changes to the Neel temperatures, *T*_N_, with substitution of rare-earth ions with Fe in SmFe_*x*_Cr_1−*x*_O_3_. We discuss the strength of the symmetric and antisymmetric Cr(Fe)–Cr(Fe) exchange interaction (*J* and *D*) effects with structural deformation and tilting, caused by changes in the average B–O–B bond angle and bond lengths.

## Experimental

Polycrystalline samples SmFe_*x*_Cr_1−*x*_O_3_ (0 < *x* < 0.5) were compounded *via* a conventional solid-state reaction using high-purity (>99.99%) raw powders of Sm_2_O_3_, Cr_2_O_3_, and Fe_2_O_3_. Powder X-ray diffraction (XRD) measurements were performed using a Bruker D5 diffractometer using Cu-Kα radiation (*λ* = 1.5418 Å). X-ray diffraction (XRD) patterns were obtained from 10 to 120° with steps of 0.02° and a counting time of 2 s per step, using an X-ray diffractometer with Cu-Kα radiation at 40 kV and 40 mA. Raman spectroscopy measurements were taken using a 514 nm wavelength argon-ion laser. The particle size and micro-structural analyses were performed using scanning electron microscopy (SEM), and energy dispersive X-ray (EDX) analysis verified the chemical composition of SmFe_*x*_Cr_1−*x*_O_3_ (0 < *x* < 0.5). Magnetization data were collected using a Quantum Design Magnetic Property Measurement System (MPMS) SQUID magnetometer over the temperature range 5–400 K. Data were collected from field-cooled cooling (FCC) measurements made over this temperature range in an applied magnetic field of 100 Oe.

## Results and discussion

### Sample characterization

**Fig. 1:**
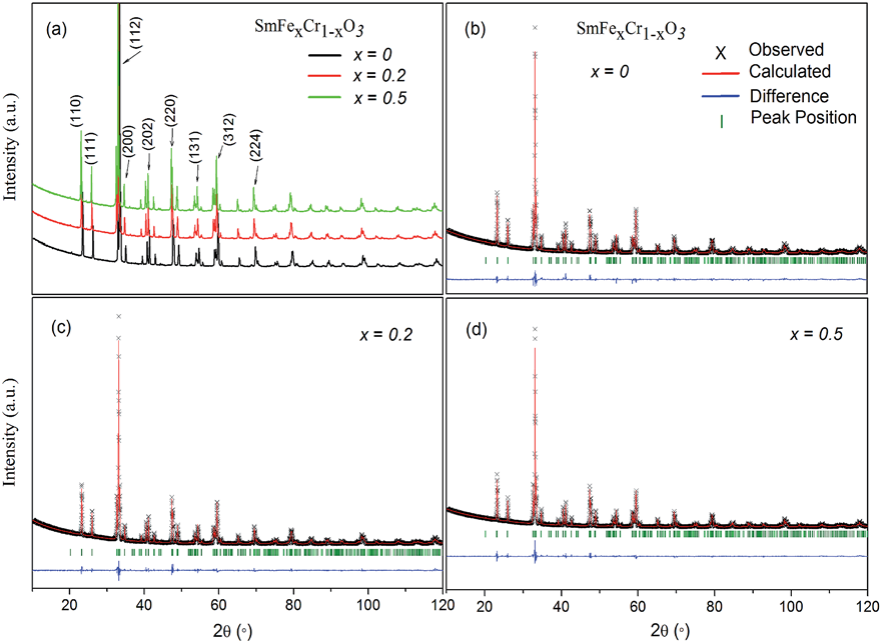
(a) The X-ray diffraction data for the SmCrO_3_, SmFe_0.2_Cr_0.8_O_3_, and SmFe_0.5_Cr_0.5_O_3_ samples. (b–d) The X-ray diffraction data and Rietveld refinement results for the SmCrO_3_, SmFe_0.2_Cr_0.8_O_3_, and SmFe_0.5_Cr_0.5_O_3_ samples.

Fig. 1 shows the XRD patterns of the samples. X-ray diffraction (XRD) patterns were obtained from 10 to 120° with steps of 0.02° and a counting time of 2 s per step, using an X-ray diffractometer with Cu-Kα radiation (*λ* = 1.5418 Å) at 40 kV and 40 mA. All the major peaks for each sample could be indexed based on an orthorhombically distorted perovskite structure with the space group *Pnma* (no. 62).^3^ Thus, these samples were determined to be phase pure within the detection limit of laboratory XRD. The lattice parameters for the first member (*x* = 0) agree well with those in the literature for samples that have been made *via* both solid-state and hydrothermal syntheses,^3,20^ and the stability of the SmCrO_3_ structure was confirmed over the temperature range 88–300 K.^21^

Rietveld refinement was used to analyze the XRD data for each composition. The Rietveld refined patterns of the SmFe_*x*_Cr_1−*x*_O_3_ (*x* = 0, 0.2 and 0.5) samples are shown in Fig. 1(b–d). The lattice parameters *a*, *b*, and *c* of the samples obtained from the Rietveld refinement are listed in Table 1. It was found that the unit cell lengths increase with increasing Fe^3+^ content, as seen in Table 1, meanwhile, the unit cell volume increases monotonically with increasing iron content. This difference could be interpreted as the effect of the dopant on the average B-site ionic radius *R*_avg_ = (x*(*R*_Fe_^3+^)^2^ + 1 − x* (*R*_Cr_^3+^)^2^)^1/2^ for the SmFe_*x*_Cr_1−*x*_O_3_ samples shown in Table 1. Here, *R*_Fe_^3+^ and *R*_Cr_^3+^ are 0.645 Å and 0.615 Å, respectively. From the Rietveld refinement, the in-plane (Cr(Fe)–O_1_–Cr(Fe)) and out-of-plane (Cr(Fe)–O_2_–Cr(Fe)) bond angles and their corresponding bond lengths were also determined (see Table 1). Note that the bond angles increase with increasing *R*_avg_. For SmCrO_3_ and SmFeO_3_, the Goldschmidt tolerance factors (*t*) are 0.8332 and 0.82046, respectively. With increasing *R*_avg_, *t* decreases for the SmCrO_3_ samples doped with Fe^3+^ at the B-site, which caused greater structural distortion.^22^ Along with the bond angles, the Cr(Fe)–O bond lengths are also slightly changed with changing Fe content, as seen in Table 1. The Cr(Fe)–O_2_ bond lies along the *b* direction of the unit cell, and both the Cr(Fe)–O_1_(1) and Cr(Fe)–O_1_(3) bonds are oriented in the *ac* plane. Increasing Fe content results in decreasing Cr(Fe)–O_2_ and Cr(Fe)–O_1_(1) bond lengths and increasing Cr(Fe)–O_1_(3) bond lengths. The overall result is that the average Cr(Fe)–O bond length slightly decreases, see Table 1. For the first member, SmCrO_3_, the values agree well with those reported previously.^3,23^

**Table 1:** The lattice parameters, average B-site ionic radius (*R*_avg_), Cr(Fe)–O bond lengths, and Cr(Fe)–O–Cr(Fe) bond and octahedral tilt angles obtained from XRD Rietveld refinement for the sample SmFe_*x*_Cr_1−*x*_O_3_ (1 < *x* < 0.5). The octahedral tilt angles *θ* and *φ* describe rotations around the [101] and [010] axes, respectively, and were calculated using the method previously reported^a^^24,25^

Sample	*X* = 0	*X* = 0.2	*X* = 0.5
*a* (Å)	5.48197	5.48841	5.52444
*b* (Å)	7.63202	7.63921	7.65942
*c* (Å)	5.36321	5.36779	5.37575
*V* (Å^3^)	224.389	225.056	227.470
*R*_avg_ (Å)	0.615	0.621	0.630
Cr(Fe)–O_1_–Cr(Fe) (deg)	147.418(0)	152.330(27)	155.710(31)
Cr(Fe)–O_2_–Cr(Fe) (deg)	146.833(1)	150.600(5)	152.300(5)
Cr(Fe)–O_1_(1) (Å)	1.99889(3)	1.95400(6)	1.95000(7)
Cr(Fe)–O_1_(3) (Å)	1.99609(3)	1.99900(6)	2.00900(7)
Cr(Fe)–O_2_ (Å)	1.99081(4)	1.97440(16)	1.97030(17)
*φ*[010]	14.3329	14.9384	16.1057
*θ*[101]	11.9479	12.0344	13.3234

a Fig. 1 The XRD patterns for La_1−*x*_Fe_*x*_TiO_3+*δ*_ (*x* = 0, 0.05, 0.1, 0.3, 0.4) at room temperature.

**Fig. 2:**
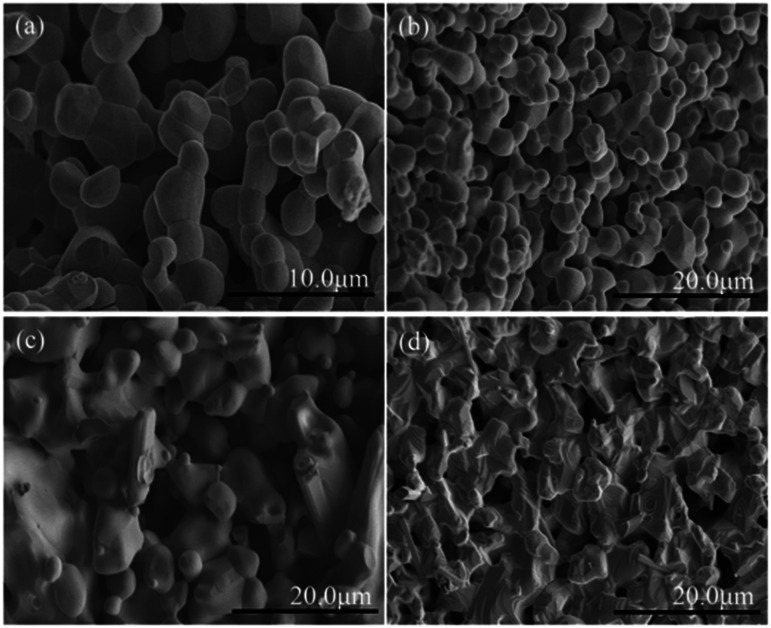
SEM images of (a and b) the as-synthesized SmCrO_3_ samples, (c) SmFe_0.2_Cr_0.8_O_3_, and (d) SmFe_0.5_Cr_0.5_O_3_.

Fig. 2 shows scanning electron microscopy (SEM) images of the as-synthesized SmFe_*x*_Cr_1−*x*_O_3_ (*x* = 0, 0.2 and 0.5) samples. Fig. 2(a and b) shows SEM images of the SmCrO_3_ samples, clearly showing a large amount of small spherical grains with a uniform distribution. The diameters of the spheres are in the range of 1 μm to 5 μm. The enlarged magnification SEM image (Fig. 2(a)) clearly shows that two grains are connected by a grain boundary. Fig. 2(c and d) shows SEM images of the SmFe_0.2_Cr_0.8_O_3_ and SmFe_0.5_Cr_0.5_O_3_ samples, respectively. From Fig. 2(b–d), it can been seen that the boundaries of the spheres become increasingly blurred with increasing Fe content, and the samples finally change to have hill-shaped morphologies, as shown in Fig. 2(d) (*x* = 0.5). In addition, the energy dispersive X-ray (EDX) spectra reveal that the relative compositional ratios of Fe to Cr atoms in the SmFe_*x*_Cr_1−*x*_O_3_ samples are about 0 : 1, 1 : 4 and 1 : 1, for the samples where *x* = 0, 0.2 and 0.5, respectively (Fig. S1–3, ESI†). The results confirmed the different Fe dopant concentrations with *x* = 0, 0.2 and 0.5 in the SmFe_*x*_Cr_1−*x*_O_3_ samples.

**Fig. 3:**
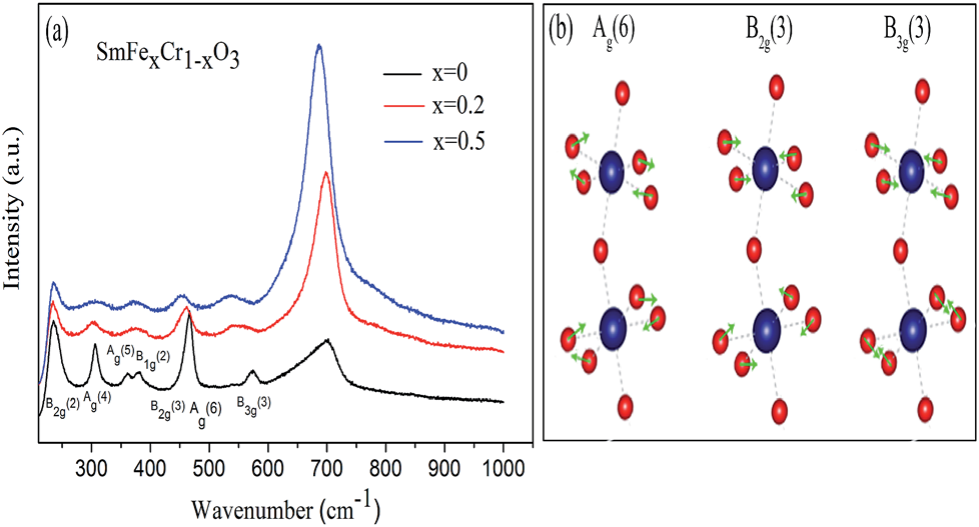
(a) Raman spectra of the SmCrO_3_, SmFe_0.2_Cr_0.8_O_3_, and SmFe_0.5_Cr_0.5_O_3_ samples. (b) Atomic-displacement patterns of the perovskite structure A_g_(6), B_2g_(3), and B_3g_(3) Raman modes.

In order to further examine the phase purity and structural characteristics of the samples, their Raman spectra were recorded at room temperature, and are shown in Fig. 3(a). The orthorhombic space group *Pnma*, distorted from an ideal cubic perovskite in which Raman scattering is formally forbidden, is predicted to have 24 Raman-active modes (7A_g_ + 5B_1g_ + 7B_2g_ + 5B_3g_) according to group theory,^26^ however, 12 modes are present within a 100–600 cm^−1^ range for the orthorhombic *Pnma* perovskite structure for RCrO_3_ systems.^27,28^ The others are either too weak in intensity or have energies below the experimental cutoff.

The positions of the Raman modes of the three samples investigated here are plotted in Fig. 3(a). In Raman scattering the frequencies of the specific lattice vibrational modes are directly related to structural distortion. The phonon modes in RCrO_3_ can be attributed to different symmetry operations: (1) those below 200 cm^−1^ are related to lattice modes involving Sm atom vibrations, and (2) the modes in the region above 200 cm^−1^ consist of various modes involving vibrations of the Sm atom and oxygen. To be specific, (1) B_1g_(1) and A_g_(3) are octahedral rotations around the crystallographic *y*-axis and A_g_(5) is a rotation around the *x*-axis (*Pnma* setting); (2) the singlet A_g_(4) is related to Sm–O vibrations; (3) A_g_(6) and B_2g_(3) arise due to bending of the CrO_6_ octahedra; (4) the B_3g_(3) mode is related to the antisymmetric stretching vibrations of the O_2_ and O_1_ atoms.^27^

Fig. 3(b) shows that the phonon frequency changes of the A_g_(6), B_2g_(3) and B_3g_(3) modes are more obvious than the others measured at room temperature. It is observed that with increasing Fe content, the phonon frequencies of the A_g_(6) and B_2g_(3) modes change from 466.01 cm^−1^ and 461.03 cm^−1^ (*x* = 0) to 461.03 cm^−1^ and 456.21 cm^−1^ (*x* = 0.2) and finally decrease to 453.56 cm^−1^ and 448.40 cm^−1^ (*x* = 0.5), respectively. The phonon frequency of the B_3g_(3) mode decreases from 573.05 cm^−1^ (*x* = 0) to 548.15 cm^−1^ (*x* = 0.2) and finally down to 533.22 cm^−1^ (*x* = 0.5). The others are almost unchanged. The changes of the mode vibration frequencies are directly related to the degree of structural distortion.^26,27^ We note that those modes discussed above show a greater dependence than others on the octahedral tilt angles *θ* and *φ*, shown in Table 1, leading to the structural distortion of the perovskite structure. This is not hard to understand: the A_g_(6) and B_2g_(3) modes are associated with the bending of the CrO_6_ octahedra, which results in rotations of the CrO_6_ octahedra in the [010] plane. Thus, the rotations lead to changes in the octahedral tilt angles *φ*.

In addition, the B_3g_(3) mode is associated with the antisymmetric stretching vibrations of the O_2_ and O_1_ atoms. Because the radius of an Fe ion is larger than that of a Cr ion, it is natural to have Raman peak shifts caused by the movement of the O_2_ and O_1_ atoms, leading to rotations of the CrO_6_ octahedra in the [101] plane.^27,29^ Therefore, the octahedral tilt angles *θ* increase with Fe content giving rise to the change of the B_3g_(3) mode. These results are in agreement with the results from the XRD data, which revealed a weak distortion of the CrO_6_ octahedra, leading to a buckling angle, by increasing the Fe doping content.

### Magnetic properties

**Fig. 4:**
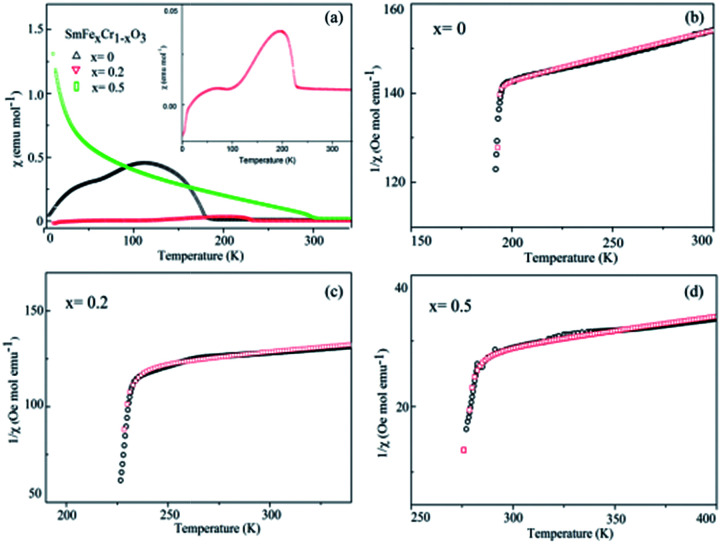
(a) The temperature dependence of the molar magnetic susceptibility (*χ*) measured in an applied field of 100 Oe for the SmCrO_3_, SmFe_0.2_Cr_0.8_O_3_, and SmFe_0.5_Cr_0.5_O_3_ samples. The inset in (a) shows the *χ vs. T* graph for the SmFe_0.2_Cr_0.8_O_3_ sample. (b–d) The temperature dependence of the inverse of the susceptibility (1/*χ*) of the SmCrO_3_, SmFe_0.2_Cr_0.8_O_3_, and SmFe_0.5_Cr_0.5_O_3_ samples, where the black circles are data points and the red circles fit to the modified Curie–Weiss law in eqn (1), which includes the DM interaction.

The temperature dependence of the magnetization of the pure and rare-earth ion substituted SmCrO_3_ samples in a measuring field *H* = 100 Oe for the field cooled (FC) cases is shown in Fig. 4. From the *χ vs. T* graph it is evident that indeed there exist various transitions pertinent to antiferromagnetic coupling (AFM), spin re-orientation transitions (SR) and Sm^3+^ ordering.^30,31^ However, the above transitions exist at various temperature ranges. Below we mainly discuss the antiferromagnetic coupling and the strength of the symmetric and antisymmetric Cr(Fe)–Cr(Fe) exchange interaction (*J* and *D*) effects with structural deformation or tilting. From Fig. 4 it is evident that a sudden jump occurs at 193 K for the compound where *x* = 0 , which can be attributed to the antiferromagnetic ordering of the Cr^3+^ moments in SmCrO_3_.^3,20^ The increase in the magnetization below this transition indicates a weak ferromagnetic moment (WFM), which arises due to the canted nature of the Cr^3+^ moments. With increasing Fe content, the samples behave as weak ferrimagnets for all concentrations of *x* below this transition temperature (*T*_N_) as shown in Fig. 4(b and c). The Neel temperature (*T*_N_) is varied in a sequential manner for the SmFe_*x*_Cr_1−*x*_O_3_ (0 ≤ *x* ≤ 0.5) compounds.^32^ The *χ vs. T* graphs demonstrated *T*_N_ values of 193 K, 228 K, and 285 K for the compounds in which *x* = 0, 0.2, and 0.5, respectively. Such a variation of *T*_N_ with Fe content could be due to the smaller ionic radius of Cr^3+^ (0.615 Å) in comparison to that of Fe^3+^ (0.645 Å). In this work, since we substituted Cr^3+^ for Fe^3+^, there is a contraction of the lattice and hence a distortion in the crystal structure. The variation of the lattice parameters with Fe^3+^ addition has been confirmed using XRD and Raman spectroscopy, measured by our group and reported elsewhere.^33^

According to the effect of t–e hybridization reported by Zhou *et al.*^34^ the change in *T*_N_ was shown to be strongly dependent on: (1) the Cr(Fe)–O–Cr(Fe) bond angle and (2) the t–e orbital hybridization introduced by intrinsic distortions in orthorhombic RCrO_3_ and RFeO_3_ perovskites. The relationship between the superexchange coupling *J*^a^ and the superexchange angle (the Cr(Fe)–O–Cr(Fe) bond angle) for the RFeO_3_ and RCrO_3_ families was based on the formula *J*^a^ ∼ cos^4^(*ω*/2)/*d*^7^,^35^ where *d* and *θ* are the B–O bond length and the averaged Cr(Fe)–O–Cr(Fe) bond angle, and *ω* = 180 − *θ*. In addition, the symmetric Cr(Fe)–Cr(Fe) exchange interactions *J* were obtained by fitting the experimental data to the modified Curie–Weiss (CW) law,^12,13^ as shown in Table 2. The values of the Cr(Fe)–Cr(Fe) exchange interactions *J* obtained from fitting the experimental data, and the *J*^a^ determined using the formula above, were investigated here and compared in Table 2. It was revealed that the change in *J* was almost in line with that of *J*^a^, and the difference between the two could be attributed to the t–e hybridization, as explained in more detail later. From the above, we believe that the increase in *T*_N_ with decreasing Cr^3+^ content is not only due to the weakening of the Fe (Cr)–O–Fe(Cr) AFM exchange interaction, but is also due to the t–e hybridization as a result of the structural distortion. The octahedral-site tilting not only reduces the t-orbital overlap integral that is considered but also introduces orbital overlap between the π (t^3^–O–t^3^) and the σ (e^2^–O–e^2^) bonds. However, the influence on the overall exchange interaction of the intersite t–e orbital hybridization is very different in Fe^3+^: t^3^ e^2^*versus* Cr: t^3^ e^0^. For RFeO_3_, t–e orbital hybridization doesn’t give any extra magnetic phase component to the overall superexchange interaction. On the other hand, in RCrO_3_, t–e hybridization gives a ferromagnetic (FM) component in addition to the existing AFM interaction in the t^3^–O–t^3^ orbitals though a superexchange interaction.^34–36^ As there exist both FM and AFM competing interactions due to t–e hybridization in SmFe_*x*_Cr_1−*x*_O_3_ compounds, by the substitution of Cr^3+^ for Fe^3+^, the strength of the FM interactions increases and the AFM interactions diminish, which may result in the increase of *T*_N_. The results are in good agreement with the report by Kotnana *et al.*^18^

**Table 2:** The magnetic parameters: the Cr(Fe) ordering temperature *T*_N_ (K), Weiss temperature *θ* (K), Curie constant *C* (emu K Oe^−1^ mol^−1^), the fitting parameter *T*_0_ (K), the symmetric exchange constant *J* (K), and the antisymmetric exchange constant *D* (K) resulting from the modified Curie–Weiss fitting of the inverse susceptibility data. The effective magnetic moment *μ*_eff_ (*μ*_B_) is calculated using the equation^3^*μ*_eff_^2^ = 3*k*_B_*C*/*N*_A_*μ*_B_^2^. *J*^a^ determined from the formula related to the superexchange angle and the displacement of oxygen ion dO (Å) obtained from the trigonometric identities

Samples	*X* = 0	*X* = 0.2	*X* = 0.5
*T*_N_ (K)	193.45(6)	228.32(4)	284.75(3)
*T*_0_ (K)	192.76(4)	227.83(3)	284.64(3)
*J*/*k*_B_ (K)^a^^3/2^	12.851(7)	15.189(5)	18.976(6)
*D*/*k*_B_ (K)^a^^3/2^	2.175(10)	1.993(10)	1.065(10)
*J*/*k*_B_ (K)^b^^5/2^	5.507(7)	6.509(5)	8.133(6)
*D*/*k*_B_ (K)^b^^5/2^	0.932(10)	0.854(10)	0.456(10)
*J* ^a^	6.72 × 10^−3^	7.50 × 10^−3^	7.65 × 10^−3^
*T*_N_ − *T*_0_	0.69(5)	0.49(4)	0.11(3)
*θ*	−1102(7)	−824(6)	−276(7)
*C*	7.9(4)	8.8(2)	14.2(3)
*μ*_eff_	7.95(10)	8.82(9)	10.66 (10)
*μ*_eff_ a	3.960	4.438	5.071
dO	0.5682	0.5010	0.4898

a ^3/2^ all *J*/*k*_B_ values were calculated assuming a full spin-only moment *S* = 3/2 (Cr) on the B site.

b ^5/2^ all *D*/*k*_B_ values were calculated assuming a full spin-only moment *S* = 5/2 (Fe) on the B site.

Now we discuss the Dzyaloshinskii–Moriya (DM) antisymmetric exchange interaction of the SmFe_*x*_Cr_1−*x*_O_3_ samples with weak ferromagnetism. The weak ferromagnetism due to the canted nature of the Cr^3+^ (Fe^3+^) moments of the samples below this transition temperature (*T*_N_) can be explained by considering the Dzyaloshinskii–Moriya (DM) interaction, which was elaborated as a consequence of spin–orbit coupling.^13^ In most materials, the temperature dependence of the magnetic susceptibility data well above *T*_N_ can be fitted to the Curie–Weiss law. However, in the present material, near *T*_N_, the susceptibility can deviate from the behavior described by the Curie–Weiss law. This deviation was modeled by Moriya^13^ for the case of weak ferromagnets (canted antiferromagnets) to now account for the DM antisymmetric exchange interaction. According to this theory, the susceptibility in the easy-axis direction obeys the CW law, whereas the susceptibility perpendicular to the easy axis must account for the DM interaction. Since the present samples are powdered (polycrystalline) samples, it is not possible to independently measure parallel and perpendicular susceptibilities, thus, the effect of the perpendicular *χ* will be dominant for the powdered sample. Therefore, we have modeled the measured powder susceptibility using eqn (1) with (*T* − *T*_0_)/(*T* − *T*_N_) resulting from the effect of the DM interaction.^13^1
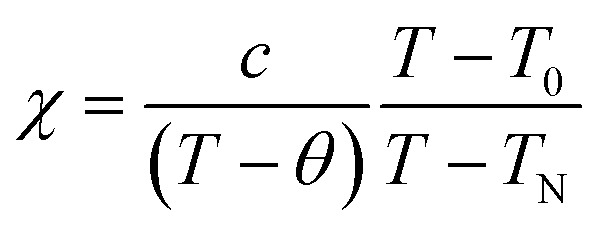
where *T* is the temperature and *T*_0_ and *T*_N_ are fitting parameters, given by2
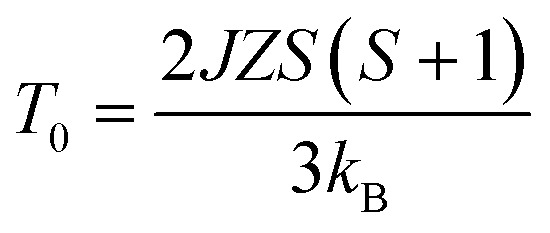
3
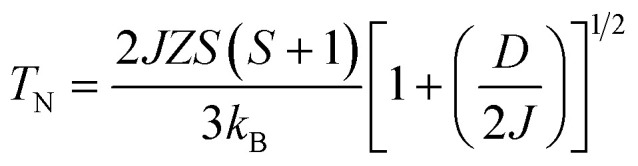
here *Z* = 6 is the coordination number of Cr^3+^ (Fe^3+^) relative to other Cr^3+^ (Fe^3+^) ions, and *S* = 3/2 and 5/2 are the spin quantum numbers of Cr^3+^ and Fe^3+^, respectively. Eqn (2) and (3) give semiquantitative analyses of *J* and *D*, the magnitudes of the symmetric and antisymmetric exchange interactions, respectively. The Cr(Fe)–Cr(Fe) exchange interactions can be extracted from the above parameters, as *T*_N_ is far above the rare-earth ordering temperature where it is possible to ignore other exchange interactions (Sm^3+^–Sm^3+^ and Sm^3+^–Cr^3+^(Fe^3+^)).

For the data for each sample above *T*_N_, the data of the inverse *χ vs. T* was fitted to eqn (1); the fitting is shown in Fig. 4 and the evaluated parameters are listed in Table 2. The Weiss constants obtained from fitting to the aforementioned modified Curie–Weiss law are negative for each of the present samples, indicating AFM ordering as expected. The effective magnetic moment *μ*_eff_ (*μ*_B_) is calculated using the equation^3^

4 *μ*_eff_^2^ = 3*k*_B_*C*/*N*_A_*μ*_B_^2^

where *N* is Avogadro’s number. Alternatively, the effective magnetic moment *μ*_eff_^*a*^ can be calculated using the free ionic moments of Sm^3+^ (0.84 *μ*_B_), Fe^3+^ (5.92 *μ*_B_), and Cr^3+^ (3.87 *μ*_B_).

5 *μ*_eff_^*a*^ = [*μ*_Sm_^2^ + (*x*)*μ*_Fe_^2^ + (1 − *x*)*μ*_Cr_^2^]^1/2^

The value of *μ*_eff_ is much higher than the theoretical value of *μ*_eff_^*a*^ (shown in Table 2) and an *ab initio* calculation suggested that the pressure produced by the tilting of the oxygen octahedra causes the difference between *μ*_eff_ and *μ*_eff_^*a*^.^37,38^ These results are in agreement with the results of XRD and Raman spectroscopy, which revealed a weak distortion of the CrO_6_ octahedra by increasing the Fe doping content. In addition, the evaluated magnetic parameters obtained in other work^3,20,37–39^ are given in the ESI (Table S1†). The magnetic parameters (*T*_N_ and *μ*_eff_) of the as-synthesized SmCrO_3_ agree well with those^3,38^ in the literature.

From the fitting shown in Fig. 4 and the evaluated parameters listed in Table 2, it is noted that the effect of the (*T* − *T*_0_)/(*T* − *T*_N_) term in eqn (1) is important only near *T*_N_, resulting in the sharp drop in the inverse *χ*, because the difference between *T*_N_ and *T*_0_ is less than 1°. The above analysis shows that for *S* = 3/2 (*S* = 5/2) the antisymmetric exchange constant *D*/*k*_B_ is 2.175 K (0.9322 K), 1.993 K (0.8543 K), and 1.06 K (0.4563 K), respectively, for the SmCrO_3_, SmFe_0.2_Cr_0.8_O_3_, and SmFe_0.5_Cr_0.5_O_3_ samples discussed here. The results show that the antisymmetric interaction *D* slightly decreases with Fe content, and the difference between *T*_N_ and *T*_0_ could well be relevant to the exchange constant *D*/*k*_B_, as seen in Table 2.

**Fig. 5:**
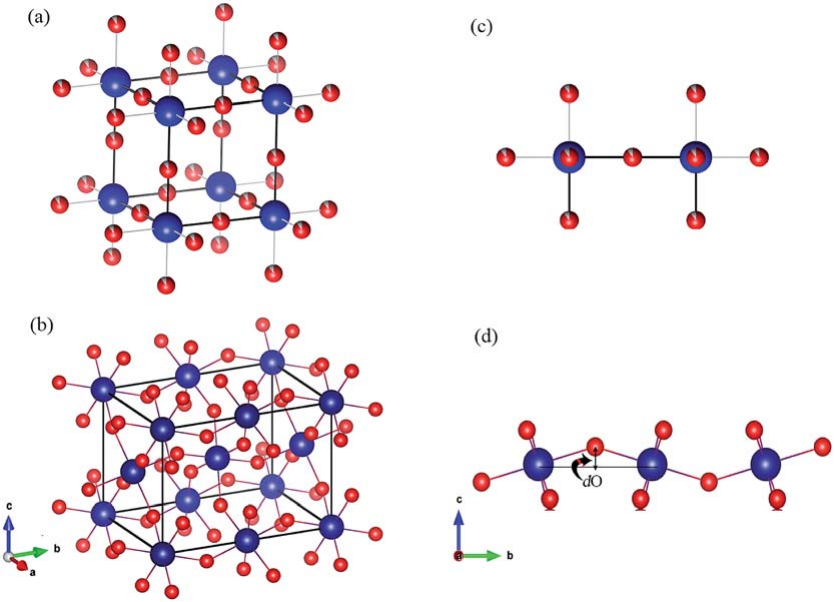
(a and b) The crystal structures of an ABO_3_ perovskite where blue = B and red = O. (a) An ideal cubic perovskite. All the nearest-neighbor B–O–B bonds are straight. (b) An orthorhombic perovskite lattice, which is the result of a size mismatch between the A and B ions. All the nearest-neighbor B–O–B bonds are bent. (c and d) Representative B–B axes from the crystal structures in (a) and (b), respectively. The dO is the displacement of the oxygen ion away from the middle point. The illustrations were created using VESTA.^45^

Moreover, according to the theory presented by Sergienko and Dagotto,^15^ the size mismatch between A and B ions usually makes the oxygen octahedra tilt and rotate, resulting in a distortion. Therefore, each oxygen ion sandwiched between two neighboring B ions may move away from the middle point, giving a bent B–O–B bond and breaking the B–B axis rotation symmetry. This bent B–O–B bond will induce a DM interaction between magnetic B ions; this is shown schematically in Fig. 5 and can be expressed as *H*_DM_ = 
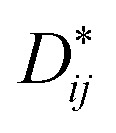
(*S*_*i*_ × *S*_*j*_), where *D*_*ij*_ is the coefficient of the DM interaction between spins *S*_*i*_ and *S*_*j*_. For a perovskite structure with bent B–O–B bonds, the vector *D*_*ij*_ must be perpendicular to the B–O–B plane. In a first-order approximation, the magnitude of *D*_*ij*_ is proportional to the displacement of the oxygen ion (dO) away from the “original” middle point, and is defined as *D*_*ij*_ = *βe*_*ij*_ × dO, where *β* is a coefficient and *e*_*ij*_ is the unit vector pointing from site *i* to site *j*.^40–44^ Finally, we obtained the displacement of the oxygen ion (dO) away from the middle point according to the trigonometric identities, using the averaged Cr(Fe)–O bond length and the Cr(Fe)–O–Cr(Fe) bond angle determined using X-ray diffraction with Rietveld refinements, as shown in Table 2. By qualitatively studying the contrast between the exchange constant *D*/*k*_B_ and the displacement of the oxygen ion (dO), it was revealed that the change in *D*/*k*_B_ was almost in line with the change in dO, which was in good agreement with the theory of Sergienko and Dagotto mentioned previously.

## Conclusions

In summary, polycrystalline samples SmFe_*x*_Cr_1−*x*_O_3_ (0 < *x* < 0.5) were compounded *via* a solid state reaction and their structures were confirmed using XRD and Raman spectroscopy techniques, which reveal the evolution of the distorted perovskite structure with Fe substitution. Note that the bond angles increase with increasing *R*_avg_. Magnetization data reveal that the Neel temperature (*T*_N_) increases with an increase in the average B-site ionic radius, and average Cr(Fe)–O–Cr(Fe) bond angle. By fitting the temperature dependence of the magnetic susceptibility to a Curie–Weiss law modified by the Dzyaloshinskii–Moriya (DM) interaction, it was found that the strength of the symmetric interaction *J* (reflected in the changes in the Neel temperature) increases with the replacement of Cr^3+^ with Fe^3+^, which is ascribed to changes in the average Fe(Cr)–O–Fe(Cr) bond angle and bond lengths, as a result of structural distortion. Meanwhile, the strength of the antisymmetric interaction *D* slightly decreased. This was mainly attributed to the displacement of the oxygen ion (dO) away from the original middle point.

## Conflicts of interest

There are no conflicts to declare.

## Supplementary Material

RA-008-C7RA13615C-s001
