# Supplementary material for: Intrinsic structural distortion and exchange interactions in SmFexCr1−xO3 compounds
Source: RSC Adv. 2018 Feb 27;8(16):8842–8. doi: 10.1039/c7ra13615c (PMC9078590; doi:10.1039/c7ra13615c)
Supplement: RA-008-C7RA13615C-s001 [file RA-008-C7RA13615C-s001.pdf]

Electronic Supplementary Material (ESI) for RSC Advances.
This journal is © The Royal Society of Chemistry 2018

Supporting information

Intrinsic structural distortion and exchange interaction in $SmFe_xCr_{1-x}O_3$ compounds

Zhongcheng Xiang,[a] Wenping Li,[a] Yimin Cui[a,*]

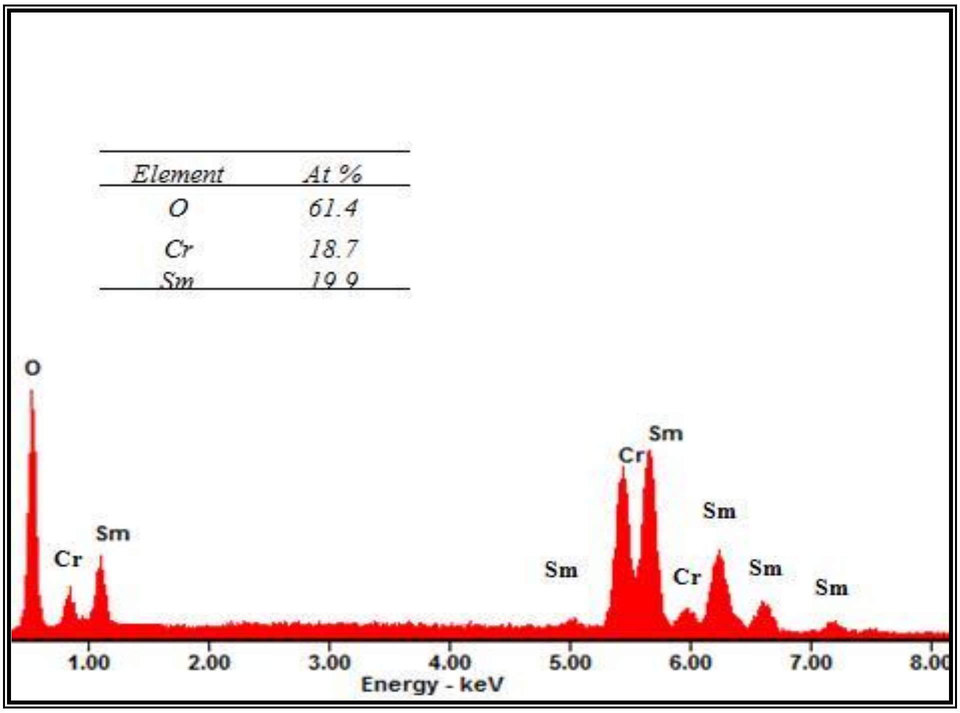

Figure S1. The original EDX spectrum of the $SmCrO_3$ samples.

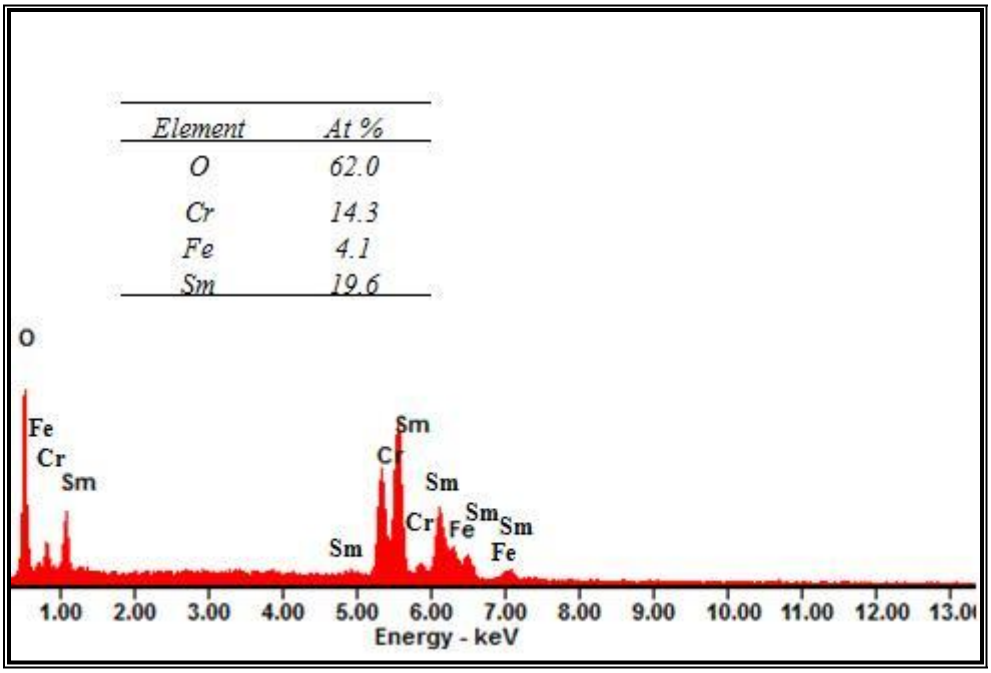

Figure S2. The original EDX spectrum of the $SmFe_{0.2}Cr_{0.8}O_3$ samples.

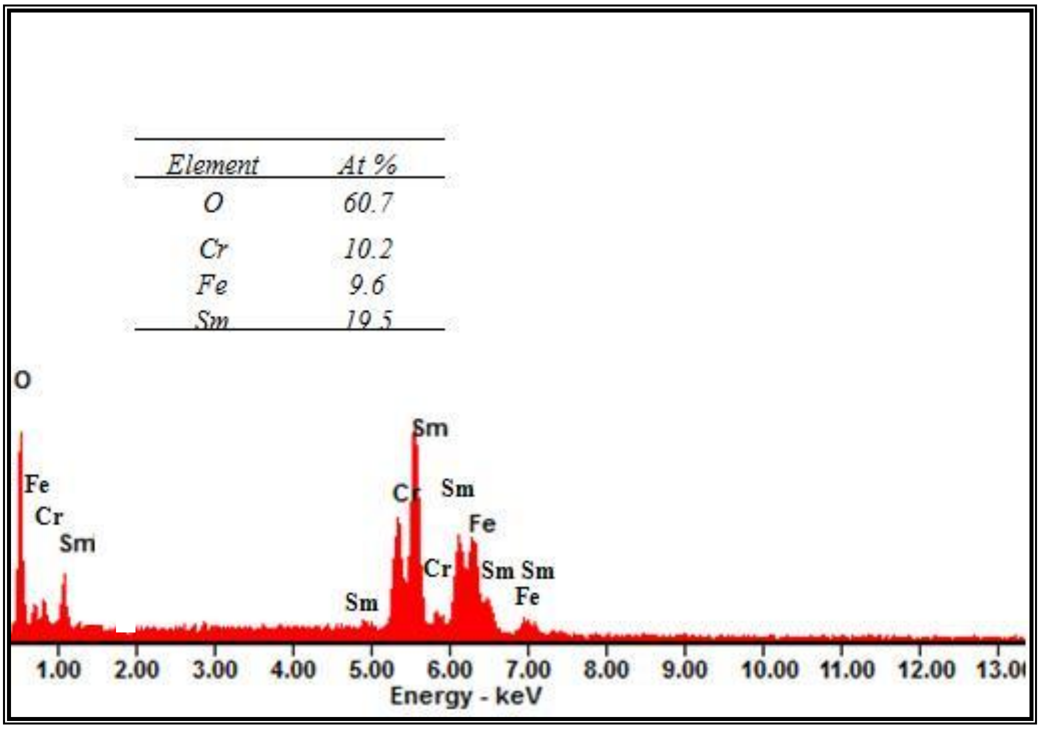

Figure S3. The original EDX spectrum of the $SmFe_{0.5}Cr_{0.5}O_3$ samples.

Table S1 Comparison of magnetic parameters for $SmCrO_3$ samples with other works.

sample	Neel temperature (K)	M_{eff} (μ_B)	Weiss temperature (K)	Ref.
SmCrO3	192	7.711	-613.4	[3]
SmCrO3	193	7.1	-1080	[38]
SmCrO3	197	-	-	[39]

$SmCrO_3$	192	5.46	-670	[20]
$SmCrO_3$	193	7.95	-1102	This work
